# Supplementary material for: Global thermal spring distribution and relationship to endogenous and exogenous factors
Source: Nat Commun. 2022 Oct 26;13:6378. doi: 10.1038/s41467-022-34115-w (PMC9606316; doi:10.1038/s41467-022-34115-w)
Supplement: Supplementary file 1 — Supplementary Information [file 41467_2022_34115_MOESM1_ESM.pdf]

Supplementary Information of

Global thermal spring distribution and relationship to endogenous and exogenous factors

Tamburello G.[1], Chiodini G.[1], Ciotoli G.[2,3], Procesi M.[3], Rouwet D.[1], Sandri L.[1], Carbonara N.[4], Masciantonio C.[4]

[1] Istituto Nazionale di Geofisica e Vulcanologia, sezione di Bologna, Bologna, Italy

[2] Consiglio Nazionale delle Ricerche, Istituto di Geologia Ambientale e Geoingegneria, Rome, Italy

[3] Istituto Nazionale di Geofisica e Vulcanologia, Rome, Italy

[4] Università degli Studi di Bologna, Bologna, Italy

Example of georeferencing of figure 16 from Waring[13] of Argentina (Supplementary Fig. 1). The lower latitudes generate a distortion that is corrected by fitting the X_P-Y_P-longitude and X_P-Y_P-latitude points (where X_P and Y_P are the pixel coordinates of known geographical features) with a Thin Plate Spline (TPS) algorithm. After georeferentiation, the locations of the thermal springs are manually picked to obtain longitude and latitude (purple circles).

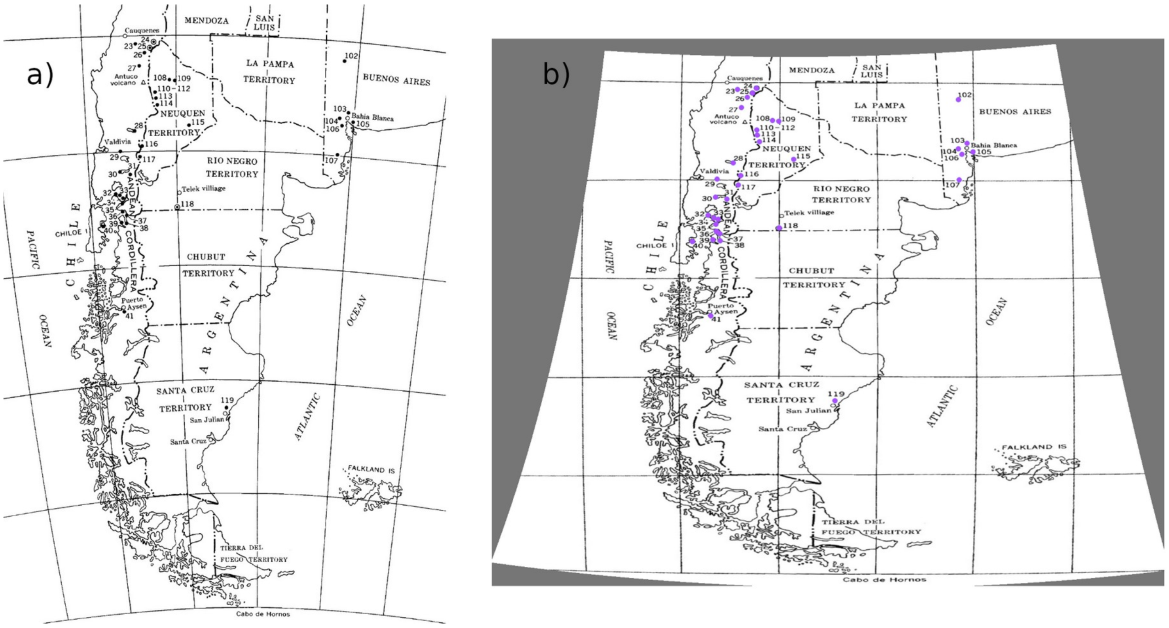

Supplementary Figure 1: Maps and thermal springs digitisation – *The map displayed in Figure 16 of Waring[13] before (a) and after (b) georeferencing. Purple circles are the manually picked thermal springs (modified after Waring[13]).*

The chemical composition of major cations and anions is reported in Waring[13] for very few thermal springs (Supplementary Fig. 2).

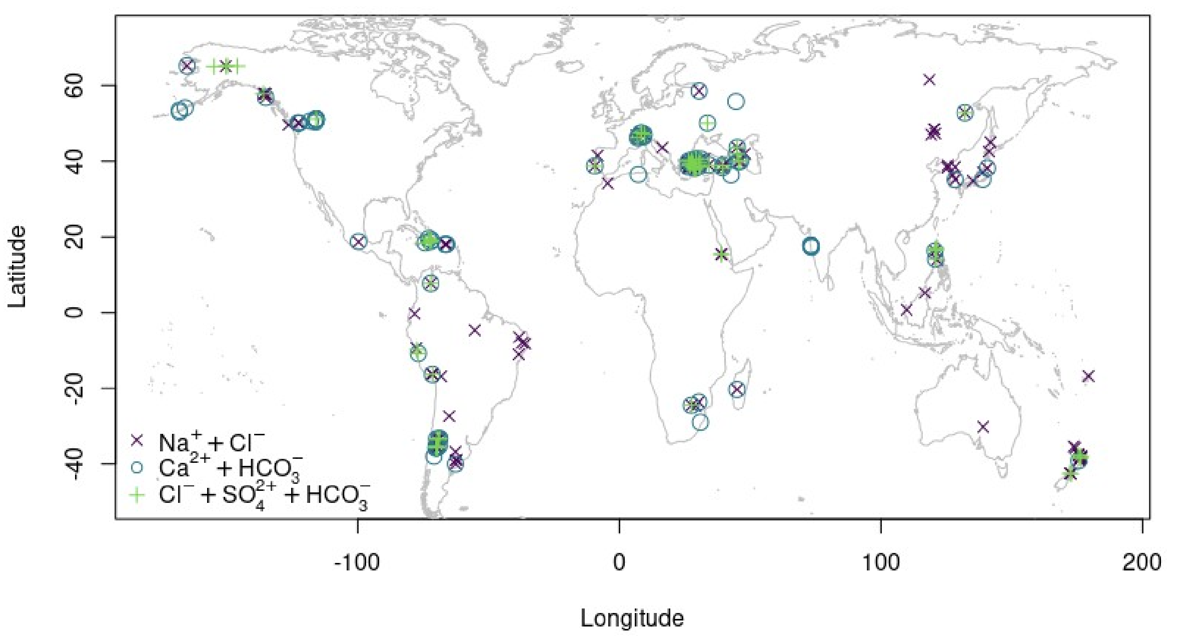

Supplementary Figure 2: Thermal springs with chemical composition - *Spatial distribution of the thermal springs with a reported chemical composition in Waring*[13] *(map created with maps*[14] *R package).*

Frequency histogram of calculated distances from each digitized thermal spring and the nearest obtained from Minissale et al.[10] and Ferguson and Graspy[11] (Supplementary Fig. 3). The vertical purple dashed line is the median value of 14 km. The high values correspond to thermal springs reported in Waring[13] but are missing in the more recent dataset (e.g., the springs in Colorado, New Mexico and Arizona that are missing in Ferguson and Graspy[11]).

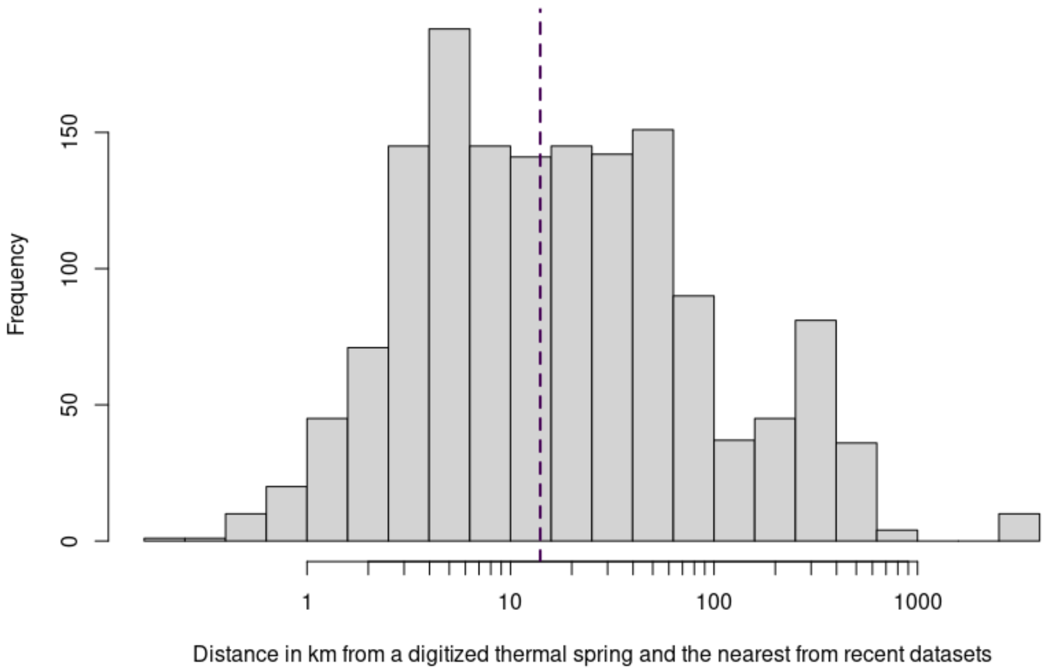

Supplementary Figure 3: Distances between thermal springs from different datasets *- Frequency histogram of distances between each digitized thermal spring from Waring[13] and the nearest thermal spring from Minissale et al.[10] and Ferguson and Grasby[11].*

Comparison between the position of the digitized thermal springs from Waring[13] and a more recent dataset for Italy[10] and North America[11]. We also compared the main clusters of thermal springs by calculating the kernel density

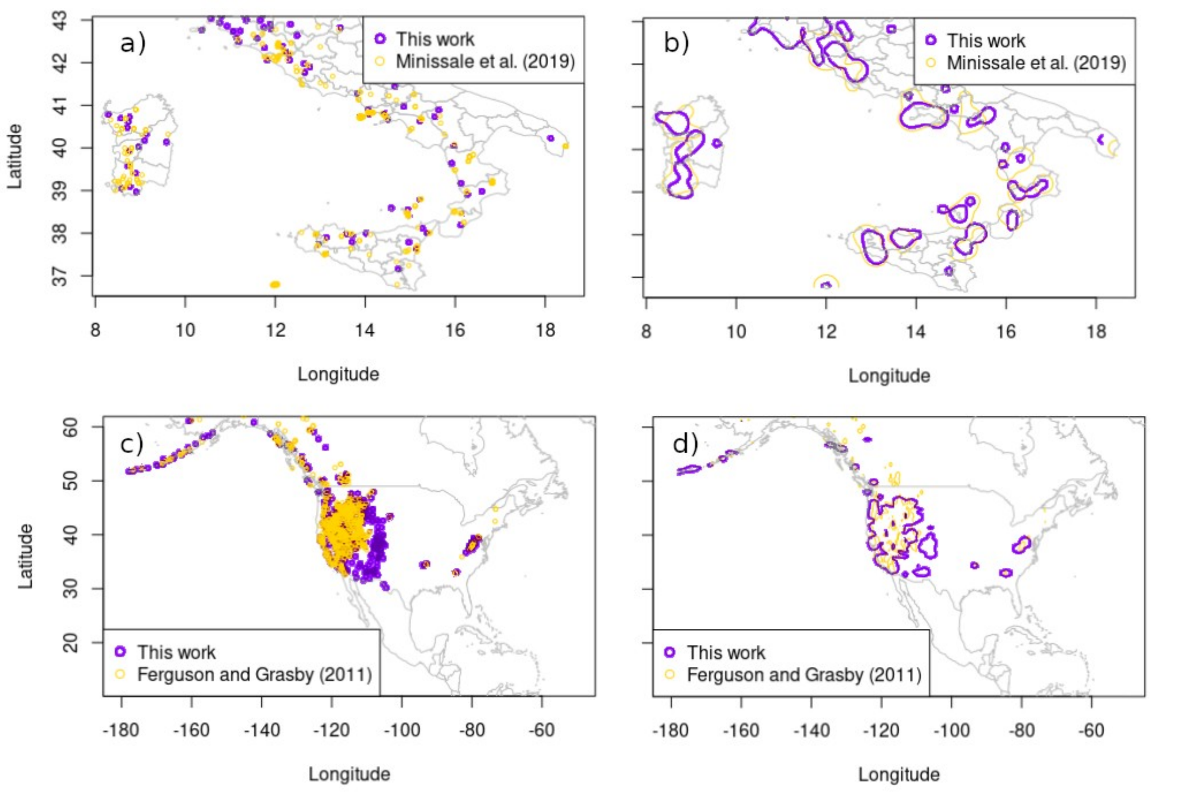

Supplementary Figure 4: Spatial distribution of thermal springs from different datasets - *Comparison between the thermal springs' distributions of Waring[13] and (a,b) Minissale et al.[10], for South Italy, and (c,d) Ferguson and Grasby[11], for North America. (b,d) Contour lines of two-dimensional kernel density estimation (map created with maps[14] R package).*

Maps showing the dataset used for the Random Forest analysis. The values per cell are reported in Supplementary data 2.

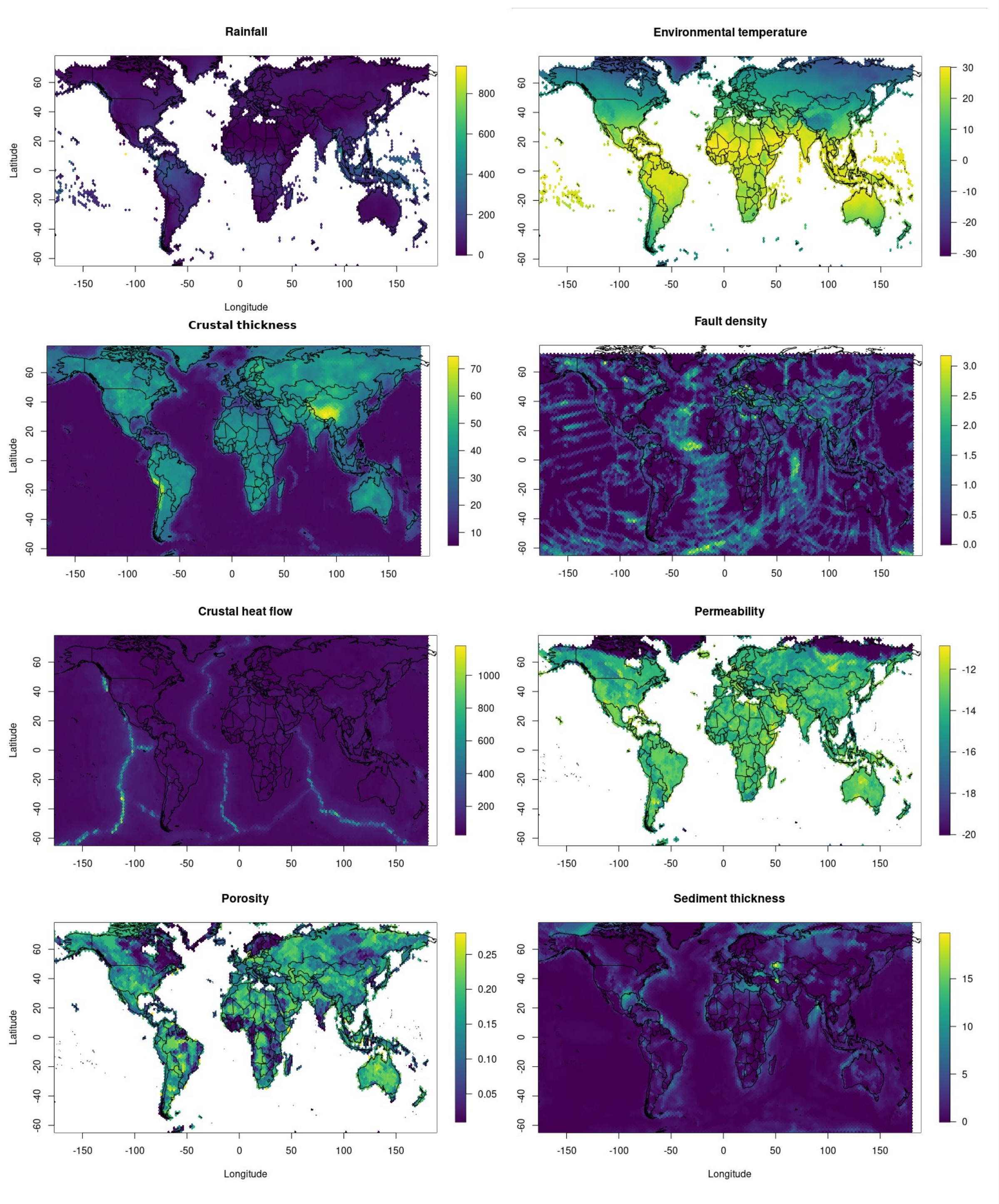

Supplementary Figure 5: Geological factors for machine learning analysis - *Maps of the input parameters used for the Random Forest Analysis (map created with maps*[14] *R package).*

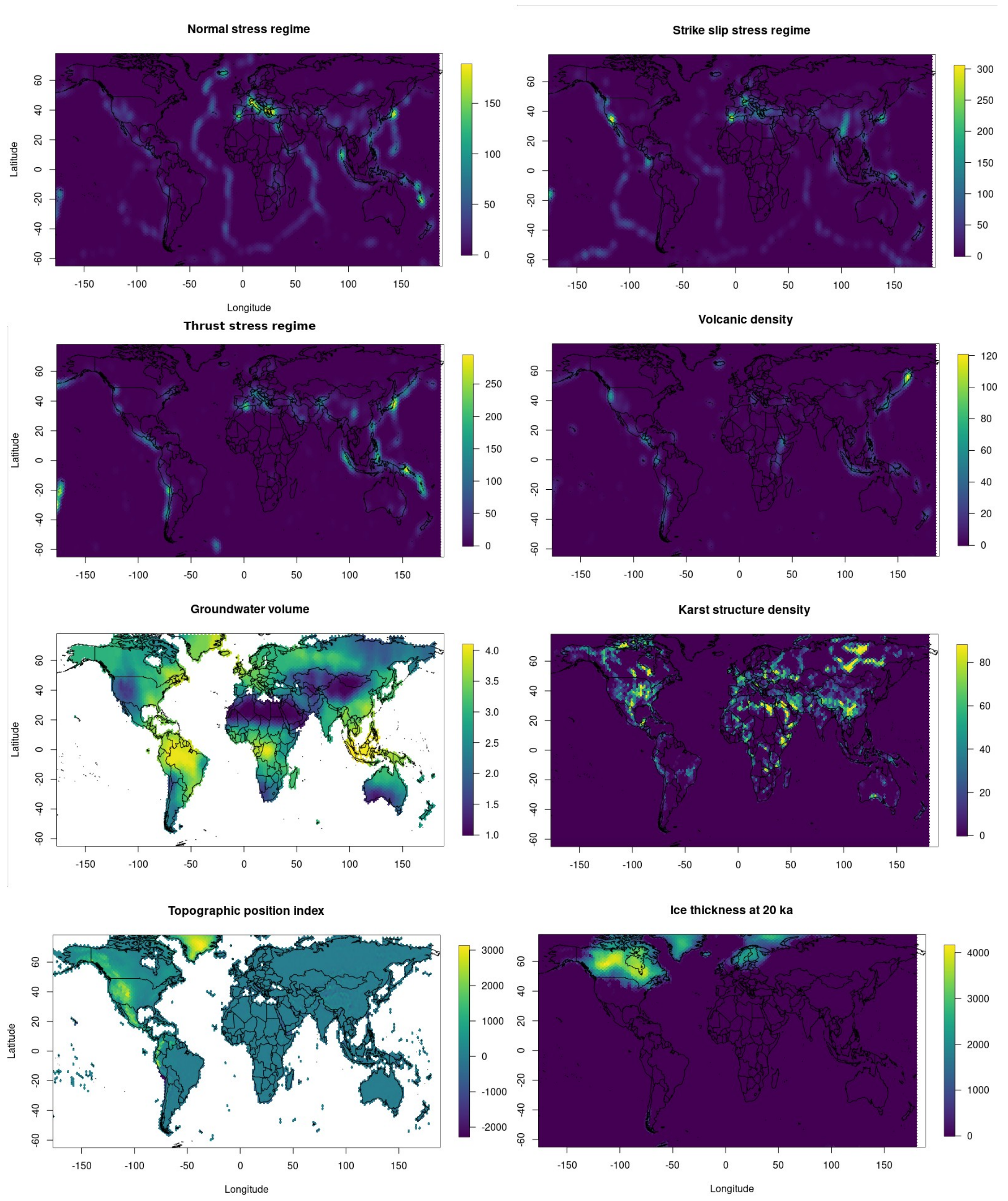

Supplementary Figure 6: Geological factors for machine learning analysis - *Maps of the input parameters used for the Random Forest Analysis (map created with maps[14] R package).*

In Supplementary Fig. 7 we show the predicted number of thermal spring areas by the random forest algorithm. The map shows a lower contrast than the prediction of the number of thermal springs (Fig. 7).

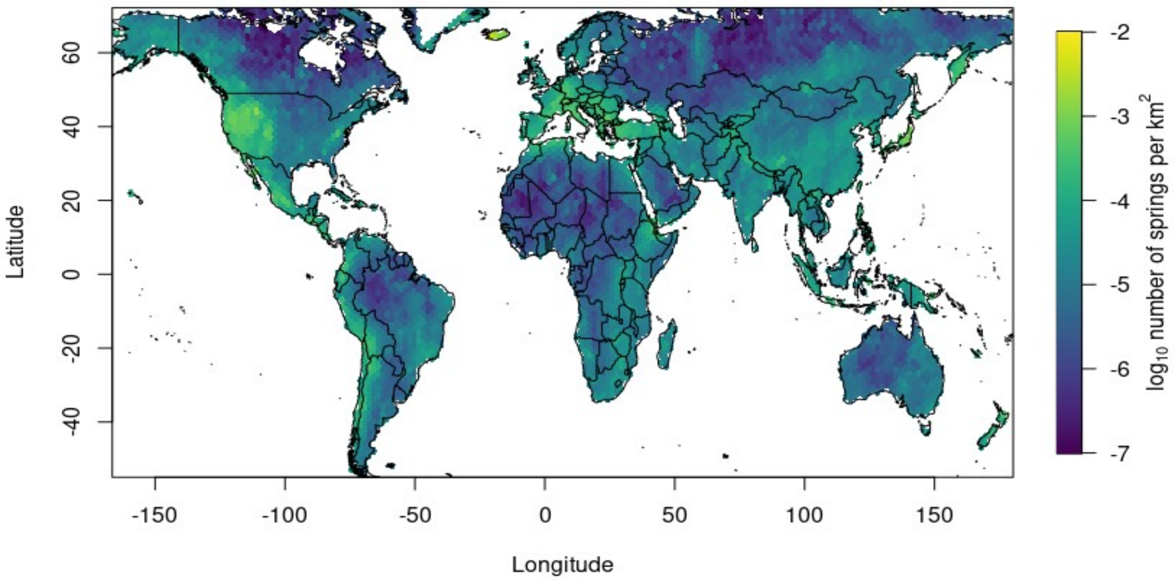

Supplementary Figure 7: Predicted number of thermal spring areas - *Random forest prediction of the distribution of the number of thermal spring areas across the globe. The value represents the averaged 500 random forest results divided by each hexagon's area in km^2 (map created with maps[14] R package).*

In the plot in Supplementary Fig. 8, we show the hexagonal areas (in grey) where there are no observed springs and the difference between the predicted and the observed number of thermal springs is significant (> 10^{-5} springs per km^2).

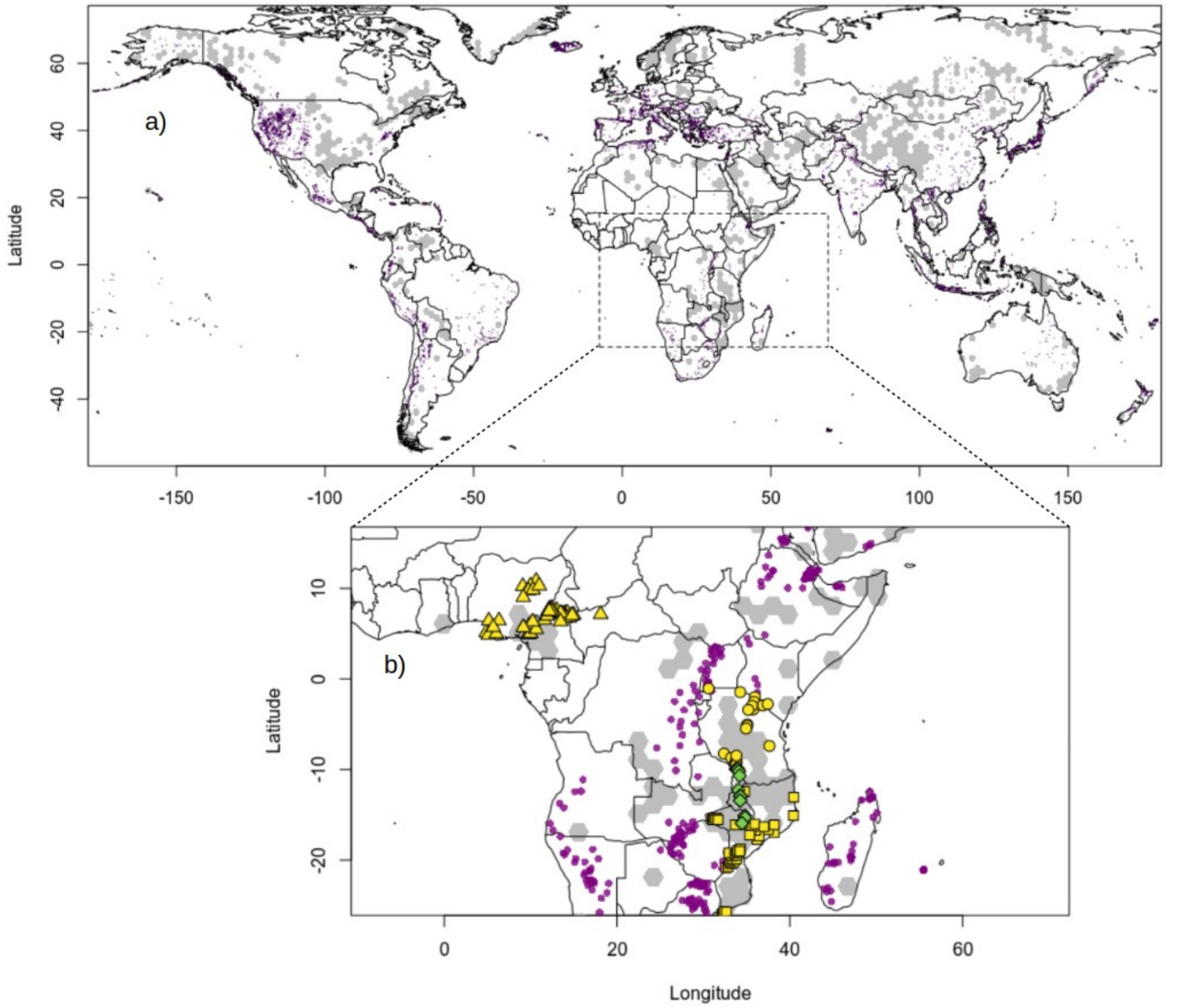

Supplementary Figure 8: Comparison between observed and predicted thermal springs – *(a) World map of the thermal springs of the world (purple circles) and (b) zoom on the African continent. The grey hexagons represent areas where the difference between the predicted and observed thermal spring densities, as log_{10} of the number of thermal springs per km^2, is > 5 and there are no observed thermal springs. We report complementary thermal springs from Cameroon[34] (yellow triangles), Tanzania[35] (yellow circles), Malawi[36] (green diamonds), and Monzambique[37] (yellow squares) (map created with maps[14] R package).*

In the subplot in Supplementary Fig. 8, we zoom on the Eastern African Rift to show how the Random Forest algorithm predicted a significant number of missing thermal springs. We searched for literature listing such missing springs and found them in Cameroon[34], Tanzania[35], Malawi[36], and Monzambique[37]. This example shows the advantages of this dataset and our analysis for future database preparations. For completeness, we list in Supplementary Data 4 the coordinates of the thermal springs added from the above literature. We suggest including these thermal springs to the dataset available in Supplementary Data 1 for future studies on thermal springs' global distribution.
