# Supplementary material for: Global thermal spring distribution and relationship to endogenous and exogenous factors
Source: Nat Commun. 2022 Oct 26;13:6378. doi: 10.1038/s41467-022-34115-w (PMC9606316; doi:10.1038/s41467-022-34115-w)
Supplement: Supplementary file 7 — Description of Additional Supplementary Files [file 41467_2022_34115_MOESM7_ESM.docx]

**Description of Additional Supplementary Files**

**File Name: Supplementary Data 1**

**Description:** Thermal springs of the world digitised from the work of Waring (1965).

**File Name: Supplementary Data 2**

**Description:** Statistical parameters calculated for all the complementary global geological datasets for each hexagonal cell of the grid.

**File Name: Supplementary Data 3**

**Description:** Average and standard deviation of the number of geothermal sites and springs for each hexagonal cell of the grid predicted by the 500 random forest runs. Calculated variable importance for all the 500 random forest runs.

**File Name: Supplementary Data 4**

**Description:** Coordinates and references of the complementary thermal springs in Africa shown in Supplementary Fig. 8
